# *Pontibacillus* sp. ALD_SL1 and *Psychroflexus* sp. ALD_RP9, two novel moderately halophilic bacteria isolated from sediment and water from the Aldabra Atoll, Seychelles

**DOI:** 10.1371/journal.pone.0256639

**Published:** 2021-08-26

**Authors:** Avril J. E. von Hoyningen-Huene, Tabea J. Schlotthauer, Dominik Schneider, Anja Poehlein, Rolf Daniel

**Affiliations:** Genomic and Applied Microbiology & Göttingen Genomics Laboratory, Institute of Microbiology and Genetics, Georg-August University of Göttingen, Göttingen, Germany; Illinois Institute of Technology, UNITED STATES

## Abstract

*Pontibacillus* sp. ALD_SL1 and *Psychroflexus* sp. ALD_RP9 are two novel bacterial isolates from mangrove sediment and a moderately hypersaline pool on the Aldabra Atoll, Seychelles. The isolates represent two novel species were characterised physiologically and genomically. *Pontibacillus* sp. ALD_SL1 is a facultatively anaerobic yellow, motile, rod-shaped Gram-positive, which grows optimally at a NaCl concentration of 11%, pH 7 and 28°C. It is the third facultatively anaerobic member of the genus *Pontibacillus*. The organism gains energy through the fermentation of pyruvate to acetate and ethanol under anaerobic conditions. The genome is the first among *Pontibacillus* that harbours a megaplasmid. *Psychroflexus* sp. ALD_RP9 is an aerobic heterotroph, which can generate energy by employing bacteriorhodopsins. It forms Gram-negative, orange, non-motile rods. The strain grows optimally at NaCl concentrations of 10%, pH 6.5–8 and 20°C. The *Psychroflexus* isolate tolerated pH conditions up to 10.5, which is the highest pH tolerance currently recorded for the genus. *Psychroflexus* sp. ALD_RP9 taxonomically belongs to the clade with the smallest genomes. Both isolates show extensive adaptations to their saline environments yet utilise different mechanisms to ensure survival.

## Introduction

We describe two novel bacterial isolates from the Aldabra Atoll, Seychelles. They represent two novel species within the *Pontibacillus* and *Psychroflexus* genera. Samples from mangrove sediment within the lagoon (*Pontibacillus* isolate) and water from a moderately hypersaline pool on Grand Terre island (*Psychroflexus* isolate) were used for enrichment and isolation of halophilic bacteria.

The genus *Pontibacillus* belonging to the *Bacillaceae* was first described by Lim *et al*. as bacillus pertaining to the sea [1]. It harbours seven validated species, *P*. *chungwhensis* [1], *P*. *halophilus* [2], *P*. *litoralis* [3], *P*. *marinus* [4], *P*. *salicampi* [5], *P*. *salipaludis* [6], and *P*. *yanchengensis* [7]. Members of the *Pontibacillus* are Gram-positive, facultatively anaerobic, moderately halophilic, endospore-forming rods and are motile through peritrichous flagella. The isolates derive from marine-related habitats including salt farms across Asia and marine lifeforms, such as sea anemones and sea urchins [8]. They require salt for growth, which generally ranges from 0.5 to 25% (w/v) NaCl. Optimal growth was recorded between 2 and 10% (w/v) NaCl. The pH optimum ranges from 7 to 8 and optimal growth temperatures from 25 to 40°C. Members of *Pontibacillus* form white- to orange-pigmented smooth colonies with a diameter of 1 to 3 mm.

The second isolate belongs to the genus *Psychroflexus* within the family *Flavobacteriaceae*. It was isolated from the pink, lower water layer of a saline landlocked pool (Westpool D). *Psychroflexus* (meaning cold bend) was first described by Bowman *et al*. in 1989 [9], who isolated the strain *Ps*. *torquis* from Antarctic sea ice and re-classified *Flavobacterium gondwanense* [10] to *Ps*. *gondwanensis*. The genus encompasses 12 validated and two non-validated published species. *Psychroflexus* species were isolated from hypersaline to saline lakes [11, 12] and salterns in China [13], Hawaii [14], Korea [15, 16], Antarctica [17], as well as saline soil [18], cheese [19] and coastal sediments [20, 21]. Growth occurs between 0 and 20% (w/v) NaCl, a pH of 6 to 10, and -16 to 40°C, demonstrating a high diversity in temperature tolerance and global dispersal of the genus. The growth optimum is generally around 2 to 10% NaCl, pH 7 to 8, and 10 to 15°C or 25 to 30°C, depending on arctic or tropical origin. Most isolates occur within the latter temperature range, thereby differing strongly from the type strain. All isolates of this genus are orange in colour.

In this study, we present two novel species affiliated to the *Pontibacillus* and the *Psychroflexus* genus. The isolates were characterised phenotypically (i) using standard microbiological techniques. In addition, complete genomes were generated using a hybrid approach of Illumina and Nanopore sequencing. Both genomes represent the second complete genome of the corresponding genus. The genomes were used to assess the phylogenetic affiliation (ii) and the potential metabolism (iii) of the strains.

## Materials and methods

### Sampling, isolation and culture

Sampling and research permissions were granted by the Seychelles Islands Foundation (Research agreement: A26), the Seychelles Bureau of Standards (Research approval letter A0157) and the Ministry of Environment, Energy and Climate Change of the Republic of Seychelles (Non-commercial transfer of genetic materials agreement 4^th^ December 2017). *Pontibacillus* sp. ALD_SL1 and *Psychroflexus* sp. ALD_RP9 were isolated from halophile medium using mangrove sediment from the South Lagoon of the Aldabra Atoll (ALD_SL), Seychelles (9°26’34.8’’S, 46°23’30.5’’E) and water from the bottom water layer of Westpool D also known as Ronny’s Pool (ALD_RP) (9°26’40.5’’S, 46°27’6.8’’E). The water (salinity 9.9%, pH 7.9, 0.10 mg/L O_2_, 32.5°C) was sampled underneath a sharp halocline. The stratification of this pool was likely caused by the occurrence of the first rainfall, leading to a bacterial bloom in the sampled bottom layer. Prior to use, the untreated sediment was stored at -80°C and the water sample at 4°C. Liquid modified growth medium with 9% total salinity (MGM9) was prepared from 30% (w/v) concentrated saltwater stock solution (SW) as described by Dyall-Smith [22]. Briefly, the 30% SW was prepared from 240 g/L NaCl, 30 g/L MgCl_2_ ∙ 6 H_2_O, 35 g/L MgSO_4_ 7 H_2_O, 7 g/L KCl, 0.5 g/L CaCl_2_ ∙ 2 H_2_O and 0.2 g/L NaHCO_3_ in deionized water. The pH was adjusted to 7.5 using tris(hydroxymethyl)aminomethane (Tris) buffer. For 1 L of MGM9, 300 mL of 30% SW were added to 5 g/L peptone (Oxoid) and 1 g/L yeast extract and deionized water. The pH was adjusted to 7.5 using Tris buffer. For solid medium, 15 g/L BactoAgar (BD Biosciences, Franklin Lakes, New Jersey, USA) was added before autoclaving. Untreated mangrove sediment (500 mg) was thawed, homogenized, and suspended in 1 ml of sterile 9% SW. Dilution series were prepared from the sediment suspension (SL) and from 100 μl of water sample (RP). They were plated on MGM9 and incubated at 28°C in the light. Isolate ALD_SL1 was picked after two days from a sediment plate and ALD_RP9 was picked after five days from a plate with water sample. Both isolates were re-streaked at least three times to purify cultures. Pure isolates were stored at - 80°C in liquid MGM9 with 15% glycerol.

### Morphology

Cells from a one-day-old culture were negatively stained with either 0.1% phosphotungstic acid (pH 7) or 0.5% uranyl acetate and applied onto a copper grid. Their morphology was determined using a Jeol 1011 electron microscope (Eching, Munich, Germany).

### Growth experiments

Growth under differing NaCl, pH and temperature conditions were determined in an adjusted liquid MGM (nMGM). For this purpose, the SW was prepared without NaCl, which was added later in the required quantities. Salt tolerance was tested in 5% (w/v) increments of NaCl up to a concentration of 25% and 1% increments between 8 and 12%. For the determination of pH and temperature optima, the isolates were incubated in medium with 11% NaCl (*Pontibacillus* sp. ALD_SL1) and 9% NaCl (*Psychroflexus* sp. ALD_RP9). Temperature was tested between 10 and 50°C at intervals of 5°C between 20 and 40°C and 28°C instead of 30°C, and pH values between 5.5 and 10.5 at intervals of 0.5. *Pontibacillus* sp. ALD_SL1 was incubated in 11% NaCl to reflect its slightly higher salt optimum. The pH intervals were adjusted with the addition of buffers (2-morpholinoethanesulfonic acid (MES), pH 5.5 and 6.0; Tris, pH 6.5 to 9.0; 3-(cyclohexylamino)-2-hydroxypropane-1-sulfonic acid (CAPSO), pH 9.5, 10.0 and 10.5) at concentrations of 1 M. For measurement of salinity and temperature optima, pH was set to 7.5. The pH and salinity experiments were incubated at 28°C. All cultures were incubated using an Orbitron shaker (Infors HT, Einsbach, Germany) at 180 rpm. Growth under the different conditions was determined in triplicate (S1 Table). For this purpose, the optical density (OD_600_) was measured using an Ultraspec 3300 Pro photometer (Amersham Pharmacia Biotec Europe GmbH, Munich, Germany) after 28 h, when both isolates had reached the stationary growth phase. Growth under anaerobic conditions was tested by placing inoculated nMGM plates with 11% NaCl (nMGM11) into an anaerobic jar with AnaeroGen 3.5 L gas packs (Thermo Fisher Scientific, Waltham, MA, USA) to generate an anaerobic atmosphere. The plates were incubated at 28°C and monitored for colony growth for 14 days. Motility of the isolates was determined in soft nMGM11 with 3.5 g/L agar.

### Physiological characterisation

Enzyme activity and carbohydrate utilisation of both isolates was tested using the API ZYM and API 50 CHB kits (bioMérieux, Nürtingen, Germany) according to the instructions of the manufacturer with adjusted salt concentrations. ALD_SL1 and ALD_RP9 cultures were washed twice before testing and resuspended in 11% or 5% saline respectively, for application in the API ZYM kit. The CHB medium of the 50 CHB kit was supplemented with 11% NaCl. Reactions in the 50 CHB kit were recorded up to 72 h of incubation. Oxidase production was tested by applying a drop of Oxidase Reagent (bioMérieux, Nürtingen, Germany) to a Rotilabo-test disk (∅ 6 mm). After adding a colony to the disk, it was monitored for a colour change. Catalase activity was tested in the same manner, but with 3% H_2_O_2_ as reagent and monitoring for bubble development. Each test was replicated three times. Both isolates were examined by Gram-staining [23].

### Genome sequencing

Genomic DNA was extracted from isolate cultures using the MasterPure Complete DNA and RNA Purification kit and the instructions of the manufacturer (Epicentre, Madison, USA) for the extraction of DNA from cell samples with the following adjustments. Cell cultures were pelleted and washed twice in PBS before DNA extraction. Cells were lysed in Cell Lysis Solution without Proteinase K and mechanically disrupted in a FastPrep (MP Biomedicals, Santa Ana, USA) for 20 s at 4 m/s with 0.1 mm glass beads. Afterwards, 2.5 μl of Proteinase K (20 mg/mL, Biotechrabbit, Düsseldorf, Germany) were added. Genomic DNA was eluted in 50 μl of nucleic acid free water and sequenced using both Illumina and Nanopore technology. Illumina paired-end reads were generated on a MiSeq sequencer using v3 chemistry (Illumina, San Diego, CA, USA) and Nanopore sequences were generated with a MinIon (Oxford Nanopore Technologies, Oxford, England) as described previously [24].

### Bioinformatic processing and analysis

Illumina and Nanopore reads were quality-filtered using fastp v0.20.0 [25]. Nanopore long-reads were filtered with a sequence cut-off of 1,000 bp (*Psychroflexus* sp. ALD_RP9) or 500 bp (*Pontibacullus* sp. ALD_SL1). Porechop v0.2.4 [26] was used for adapter-trimming and read-splitting. Sequences were assembled with Unicyler v0.4.8 and the conservative hybrid assembly approach [27]. Initial assessment of genome relatedness and taxonomy was performed using the Genome Taxonomy Database Tool kit (GTDB-Tk) and database v1.0.1 [28]. Assembled genomes were annotated with the NCBI Prokaryotic Genome Annotation Pipeline (PGAP) v4.13 [29] and are accessible under the accessions CP062974, CP062975 and CP062973. The 16S rRNA gene consensus sequences of each genome were aligned against the 16S rRNA gene sequences of all other members of each genus with ClustalW in MEGA-X 10.1.8 [30]. MEGA-X was also used to calculate neighbor-joining, maximum-likelihood and maximum-parsimony phylogenetic trees with the Kimura two-parameter model and 1,000 bootstraps. Genome average nucleotide identity (ANI) was compared using the ANIm method in pyANI v0.2.10 [31] and similarities visualised using the Blast Ring Image Generator (BRIG) [32]. All available genome assemblies, ranging from contig via scaffold to complete, were used for ANI analysis. Cellular functions of both isolate genomes were inferred using BlastKOALA against the KEGG database [33] and pathway visualisation using the KEGG Mapper [34]. Transmembrane domains and signal peptides were predicted using TMHMM v.2.0 [35] and SignalP 5.0 servers [36]. Genomic islands and prophages were identified using IslandViewer4 [37] and PHASTER [38]. Putative antibiotic resistance genes (ARGs) were identified by searching the Resfams database v1.2.2 using HMMER 3.3 [39, 40]. ARGs detected with Resfams were additionally verified with deepARG v2.0. Further hits were added to the putative list if identified genes crossed a threshold of 50% identity, a bit score above 50 and an e-value below 1e-20 [41].

## Results and discussion

### Cell and colony morphology

**Fig 1:**
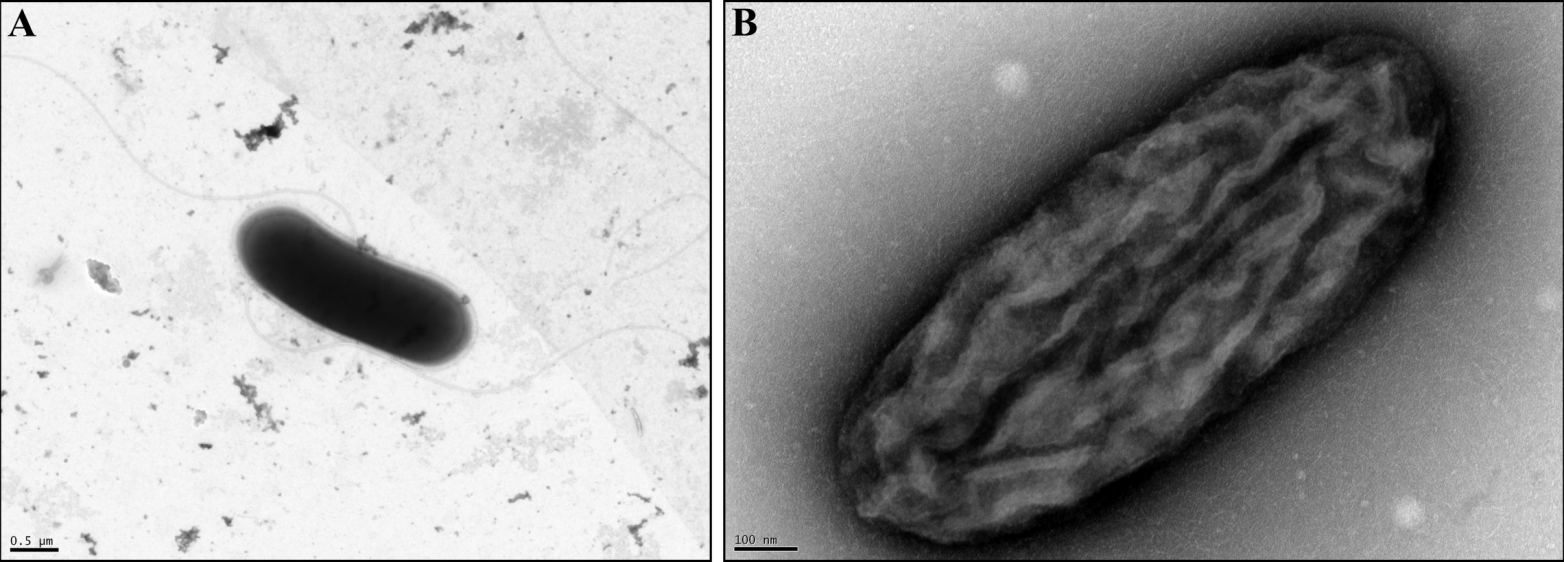
Transmission electron micrograph of *Pontibacillus* sp. ALD_SL1 (A) and *Psychroflexus* sp. ALD_RP9 (B). Cells from an overnight culture were stained with phosphotungstic acid (A) or uranyl acetate (B). A: rod-shaped *Pontibacillus* sp. ALD_SL1 with two long peritrichious flagella. B: *Psychroflexus* sp. ALD_RP9 cells are rod-shaped and lack flagella or pili. EPS are visible as a web of thin filaments broadly surrounding the cell. The scale bars in the bottom left measure 0.5 μm (A) and 0.1 μm (B).

*Pontibacillus* sp. ALD_SL1 is a Gram-positive, rod-shaped aerobic heterotroph, which forms yellow, opaque colonies with an entire margin. Cells are 2.5–3 μm x 0.8–1 μm, motile rods with peritrichous flagella (Fig 1A). *Psychroflexus* sp. ALD_RP9 is a Gram-negative, rod-shaped aerobic heterotroph. It forms orange, convex, gelatinous colonies. Cells are 1.1–1.3 μm x 0.4–0.6 μm in size, lack flagella and pili, and are surrounded by a web of exopolysaccharides (EPS), which contribute to the gelatinous texture of the colonies (Fig 1B).

### Growth kinetics and optima

Both isolates were moderately halophilic with growth matching the reported ranges for members of the corresponding genus [5, 12]. The NaCl optima were at 10% (ALD_RP9) and 11% (w/v) NaCl (ALD_SL1) (Table 1, S1 Table). ALD_SL1 maintained growth between pH values of 6 and 10 with an optimum at pH 7. ALD_RP9 showed optimal growth between pH 6.5 and 8 and diminished growth between pH 9–10.5. Growth beyond pH 10.5 was not tested due to strong precipitation of medium components above this value. ALD_SL1 grew optimally at 28°C. ALD_RP9 had a narrower growth range (20–40°C) but same optimum as other members of the genus [19]. Incubation for 14 days under an anaerobic atmosphere showed growth of *Pontibacillus* sp. ALD_SL1, indicating that it is one of the facultative anaerobes of the genus [3, 6]. *Psychroflexus* sp. ALD_RP9 showed only punctiform colonies, which may have benefited from residual oxygen at the start of the experiment.

**Table 1:** Morphological, growth and enzymatic characteristics of *Pontibacillus* sp. ALD_SL1 and *Psychroflexus* sp. ALD_RP9.

Characteristic	*Pontibacillus* sp.	*Psychroflexus* sp.
	ALD_SL1	ALD_RP9
Cell length (μm)	2.5–3	0.4–0.6
Pigmentation	yellow	orange
Gram-staining	+	-
Motility	+	-
Microaerophilic growth	+	+
Temperature range (°C)	20–40 (28)	20–40 (20)
NaCl range (%, w/v)	5–20 (11)	5–15 (10)
pH range	6–10 (7)	6–10.5 (6.5–8)
Enzyme activity:		
Oxidase	-	-
Catalase	+	-
Alkaline phosphatase	+	+
Esterase (C4)	+	+
Esterase lipase (C8)	+	+
Leucine arylamidase	-	+
Valine arylamidase	-	+
Cysteine arylamidase	-	+
Trypsin	-	+
Acid phosphatase	-	+
Naphtol-AS-BI- phosphohydrolase	-	+
α-glucosidase	+	-
Acid production:		-
Glycerol	+	-
_D-_Ribose	+	-
_D-_Glucose	+	-
_D-_Fructose	+	-
_D-_Maltose	+	+
Sucrose	+	-
_D-_Trehalose	+	-
Inulin	+	-
Starch	+	+
Glycogen	+	-
Potassium 5-ketogluconate	v	-

Temperature, NaCl and pH optima are indicated in brackets. Both strains were negative for lipase (C4), α-chymotrypsin, α/β-galactosidase, β-glucuronidase, β-glucosidase, N-acetyl-β-glucosaminidase, α-mannosidase and α-fucosidase. Unless listed, tests from the API CHB kit were negative after 72 hours. Reactions are positive (+), negative (n) or variable (v).

**Fig 2:**
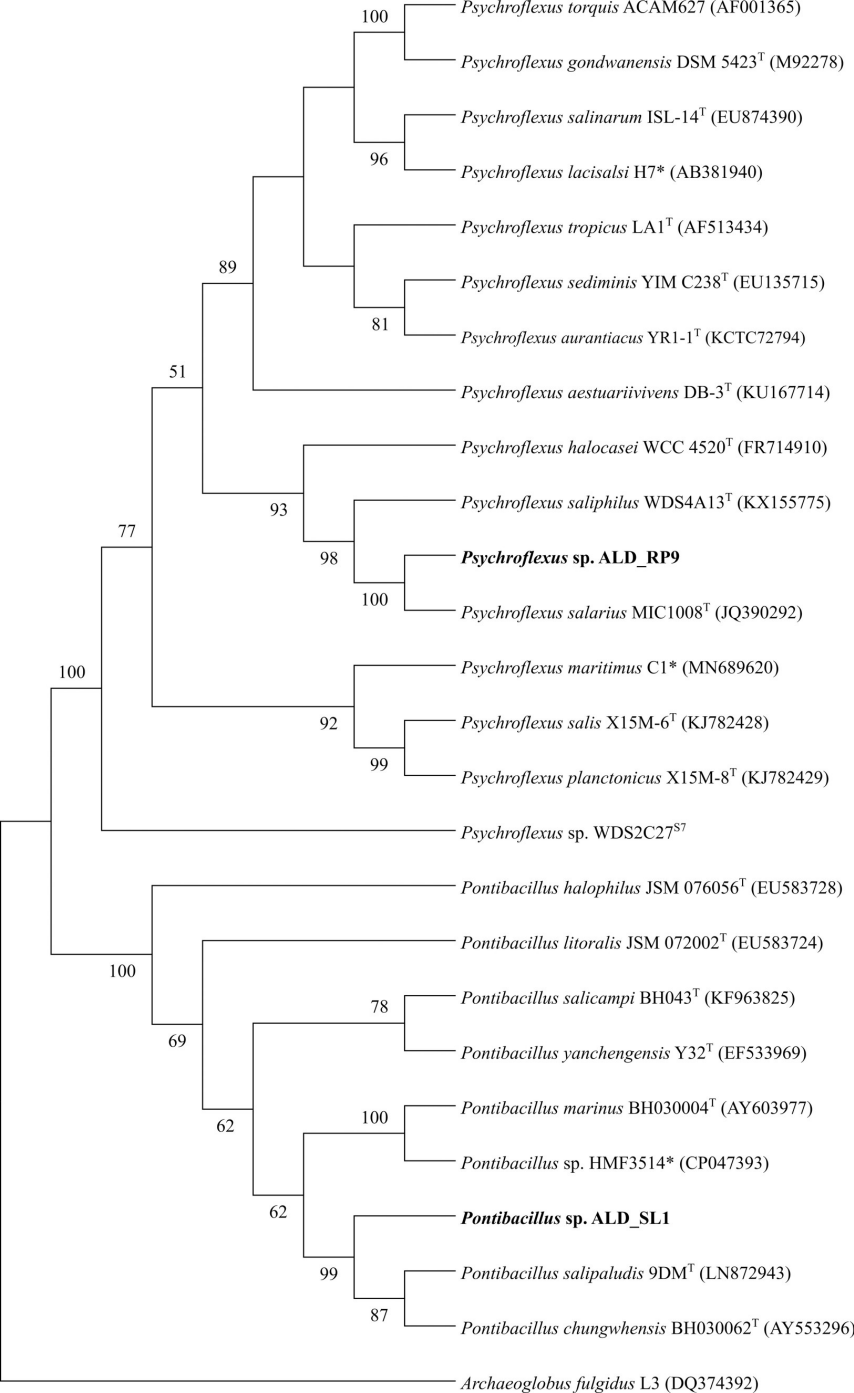
Neighbor-joining phylogenetic tree of the Pontibacillus and Psychroflexus genera. The tree includes 16S rRNA gene sequences from genomes (*), and genome scaffolds (S and scaffold number). The optimal tree with the sum of branch length 1.05278265 is shown. The percentage of replicate trees in which the associated taxa clustered together in the bootstrap test (1,000 replicates) are shown next to the branches. Bootstrapping values at the branches indicate the mean result of the neighbor-joining, maximum-likelihood and maximum-parsimony method. Evolutionary distances were calculated using the Kimura 2-parameter model with *Archaeoglobus fulgidus* L3 as an outgroup.

Both isolates were tested for activity of certain enzymes and carbohydrate metabolism using API kits ZYM and CHB. *Pontibacillus* sp. ALD_SL1 was catalase positive and oxidase negative and showed enzyme activity for four of the 19 tested substrates, namely alkaline phosphatase, esterase (C4), esterase lipase (C8) and α-glucosidase activity. Acid production was observed for 10 of the 50 tested carbohydrates, including glycerol, D-ribose, D-glucose, D-fructose, D-maltose, sucrose, D-trehalose, inulin, starch, and glycogen (Table 1). This profile indicated that ALD_SL1 is metabolically more similar to *P*. *chungwhensis* than to *P*. *salipaludis* [6], which cluster together phylogenetically (Fig 2). *Psychroflexus* sp. ALD_RP9 was both catalase and oxidase negative and hydrolysed 9 of the 19 tested substrates using alkaline phosphatase, esterase (C4) and esterase lipase (C8), leucine, valine, and cysteine arylamidase, trypsin, acid phosphatase, and napthol-AS-BI-phosphohydrolase. Metabolic tests with the CHB kit showed acid production from maltose and starch (Table 1). While the growth ranges of all *Psychroflexus* isolates are similar, results from the metabolic tests differ strongly within the genus. Interpretation of these results is hampered by inconsistencies between studies and analyses regarding preparation (with/without NaCl) and incubation times (2–10 days) [12, 19].

### Genome assembly and characteristics

**Fig 3:**
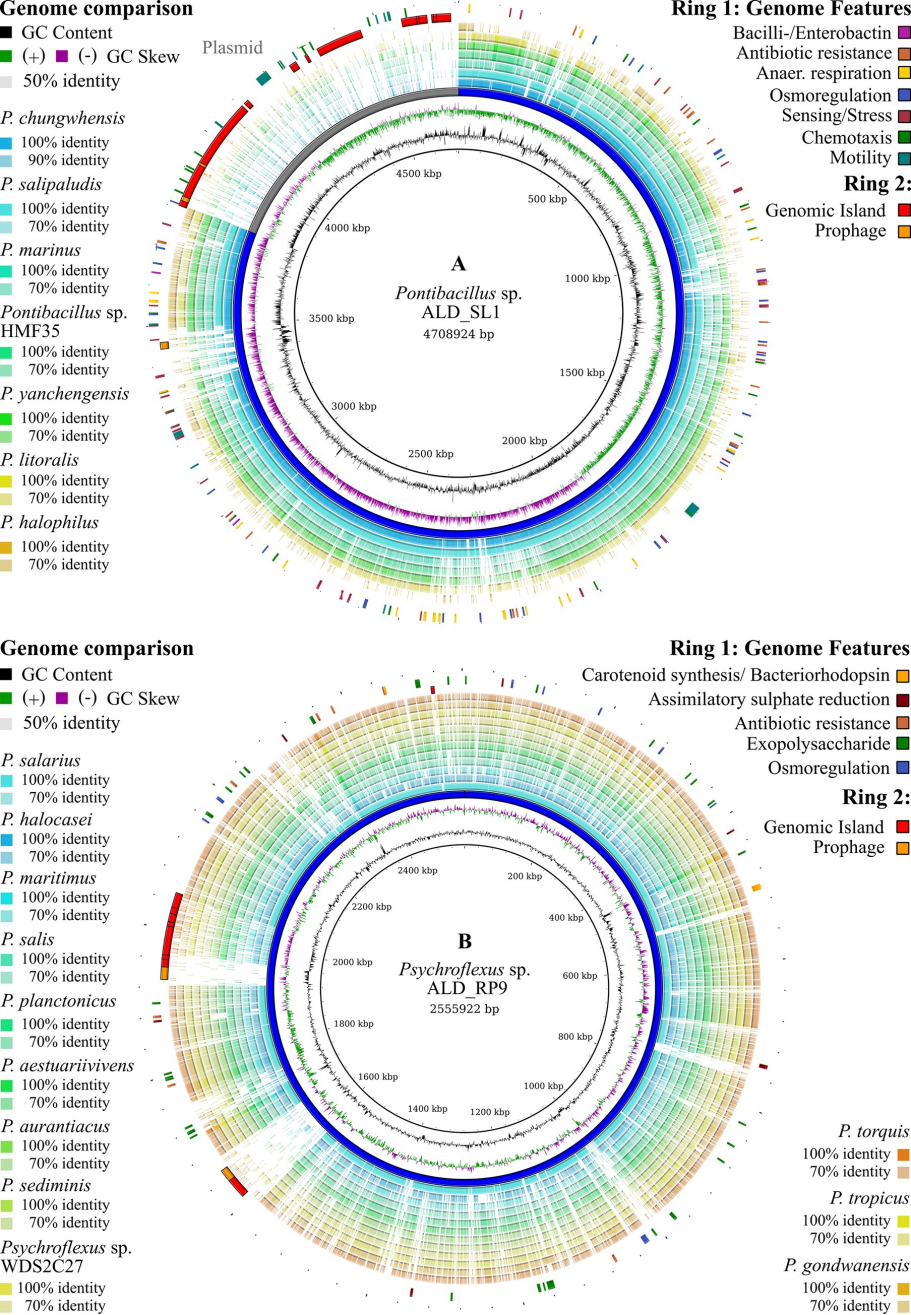
Genome features and comparison of *Pontibacillus* sp. ALD_SL1 and *Psychroflexus* sp. ALD_RP9 with all available scaffolds and genomes of their genus. The image was generated using BRIG [32] and a nucleotide blast for genome comparison. Ring 1: selected genome features from the functional annotation with BlastKOALA [33]. Ring 2: genomic islands (red) predicted with IslandViewer 4 [37] and prophages (orange) identified using PHASTER [38]. Rings 3–9 (A) and 3–11 (B): available genomes and scaffolds for each genus.

Two complete genomes were assembled with a hybrid assembly using long Nanopore reads and short Illumina paired-end reads. Quality-filtering with fastp removed 59% of the ALD_SL1 Nanopore reads, mainly due to the length constraints. Quality-filtering of ALD_SL1 Illumina reads removed 4% of the reads. The *Pontibacillus* sp. ALD_SL1 genome (CP062974) and plasmid (CP062975) were assembled from 233,024 quality-filtered Nanopore reads with a mean length of 1,438 bp and 7,554,974 quality-filtered Illumina reads with a mean length of 235 bp. The genome assembly resulted in a closed circular chromosome (3,811,075 bp) and megaplasmid (897,839 bp) with a GC content of 40.84 and 42.38%, respectively. The whole genome exhibited a mean read coverage of 443 x, 4,759 putative protein-encoding genes, 24 rRNAs, 78 tRNAs and five non-coding RNAs (Table 2). Of all sequenced *Pontibacillus* isolates, this is the only genome to harbour a (mega)plasmid. Of 1,236 hypothetical proteins in the whole genome, 725 are located on this plasmid. More than 50% of the megaplasmid were predicted as genomic islands, including two prophage regions. The chromosome harbours one putative prophage region (Fig 3).

**Table 2:** Genome compositions.

Genome characteristic	*Pontibacillus* sp.	*Psychroflexus* sp.
	ALD_SL1	ALD_RP9
Genome size	4.7 Mbp	2.6 Mbp
Extrachromosomal features	Plasmid: 897,839 bp	-
Genome coverage	443x	1,056x
GC content	41.1%	33.1%
Protein coding genes (CDS)	4,759	2,336
RNA-encoding genes	107	49
rRNA	24	9
tRNA	78	36
nc RNA	5	4
Pseudogenes	157	13
Hypothetical proteins	1,263	431
Genes with transmembrane domains	1,350	546
Genes with signal peptides	437	492
SP (Sec/SPI)	271	298
TAT (Tat/SPI)	6	1
Lipo (Sec/SPII)	160	193

Quality filtering prior to *Psychroflexus* sp. ALD_RP9 assembly resulted in the removal of 16% of Nanopore and 4% of Illumina reads. The complete circular genome (CP062973) was assembled from 119,567 Nanopore reads with a mean length of 14,283 bp and 3,909,382 Illumina reads with a mean length of 262 bp. The genome comprises a single circular chromosome (2,555,922 bp) with a GC content of 33.1%. The mean read coverage was 1,056-fold. A total of 2,336 putative protein-encoding genes were assigned of which 431 were hypothetical. In addition, genes for nine rRNAs, 36 tRNAs and four non-coding RNAs were identified. Two genomic islands were identified, which overlap with the prophage predictions. These regions share low sequence similarity or are absent from the other *Psychroflexus* genomes (Fig 3).

### Phylogeny

**Fig 4:**
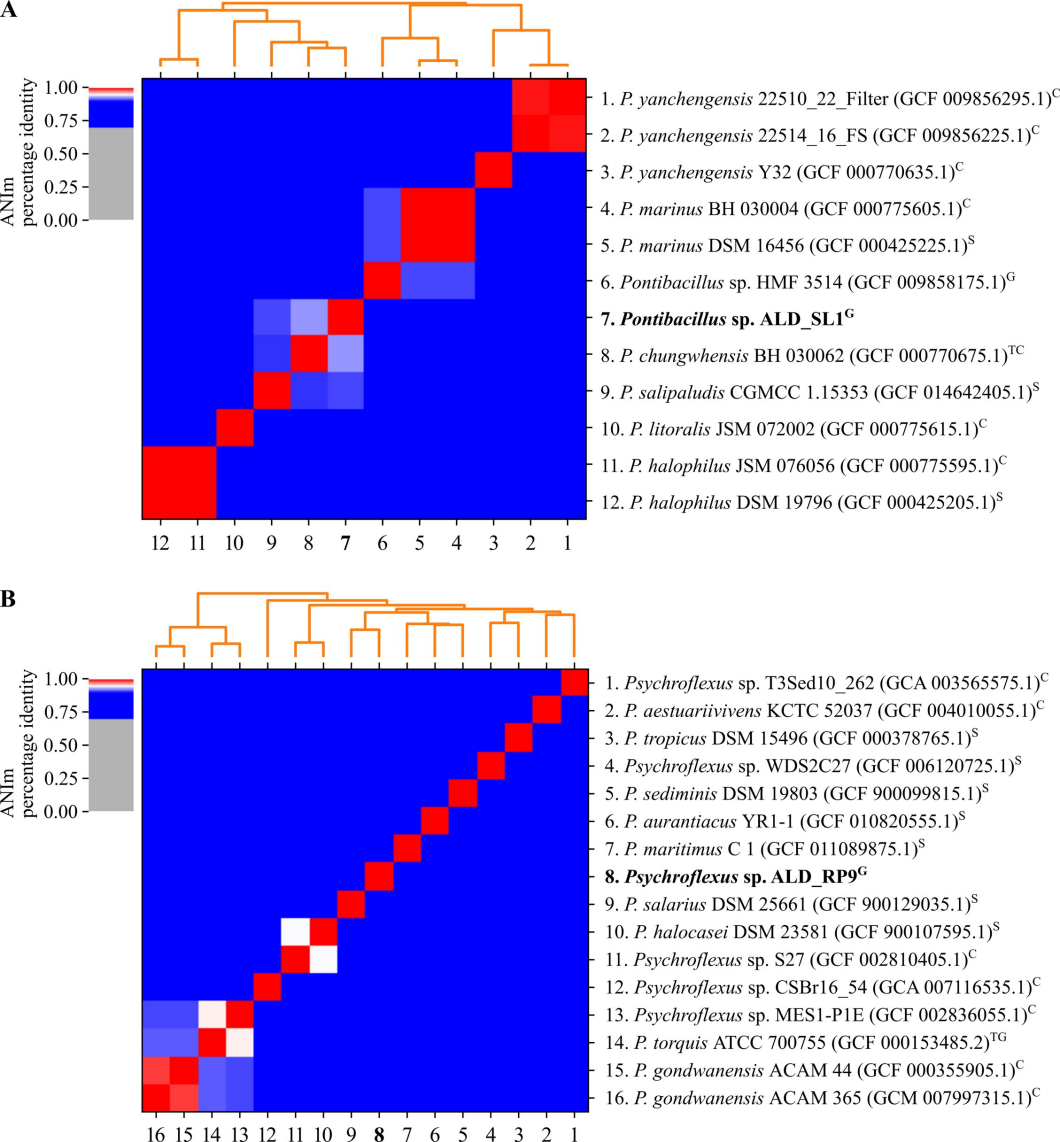
Relatedness of *Pontibacillus* (A) and *Psychroflexus* (B) genomes. All available genome assemblies of each genus were aligned and compared using ANIm in pyANI v0.2.10. [31]. The level of genome completeness is indicated in upper case: contig (C), scaffold (S), full genome (G), genus type species (T).

A neighbour-joining phylogenetic tree was built with the consensus 16S rRNA gene sequences from each isolate and the 16S rRNA genes of all available genomes and scaffolds (Fig 2). GTDB-Tk resolved their taxonomy to the genus level with a RED value of 0.995 (ALD_SL1) and 0.994 (ALD_RP9), indicating that both are new species. Relatedness on the genomic level was assessed through average nucleotide identity analysis (ANIm) with all available genomes and genome assemblies (Fig 4). The available *Pontibacillus* genomes in the NCBI database consist of seven contig-level assemblies, two scaffolds and one complete genome (*Pontibacillus* sp. HMF3514). *Pontibacillus* sp. ALD_SL1 is most closely related to *P*. *chungwhensis* BH030062^T^ (ANI 93%) and *P*. *salipaludis* CGMCC 1.15353 (ANI 91%), which were isolated from a solar saltern in Chungwha, Korea [1] and marine sediment in Tuticorn, India [6]. All other available genome assemblies remain below 90% ANI, however, these values may be exaggerated due to gaps in the unclosed genomes and scaffolds.

Available *Psychroflexus* assemblies comprise seven contig-level genomes, seven scaffolds and one complete genome (*Ps*. *torquis*). *Psychroflexus* sp. ALD_RP9 is most closely related to *Ps*. *salarius* MIC1008^T^ with an ANI of 89% and forms a well-supported cluster with *Ps*. *saliphilus* WDS4A14^T^ and *Ps*. *halocasei* WCC 4520^T^. The relation of this branch to the cluster around the type species *Ps*. *torquis* ACAM627^T^ and *Ps*. *tropicus* LA1^T^ could not fully be resolved (bootstrap values < 60) using the three tree building methods (Fig 2). Members on this branch contain the smallest genomes of the genus with an average of 2.62 Mbp. Smaller genomes and a higher percentage of coding genes (92%) than more distant relatives i.e., *Ps*. *torquis* (4.32 Mbp, 81%) [42], indicate genome streamlining [43]. However, it has been proposed that the reverse is true and *Ps*. *torquis* may have benefitted from extensive gene acquisition to survive in its native arctic environment, leading to an increased genome size [42].

### Genome features

**Fig 5:**
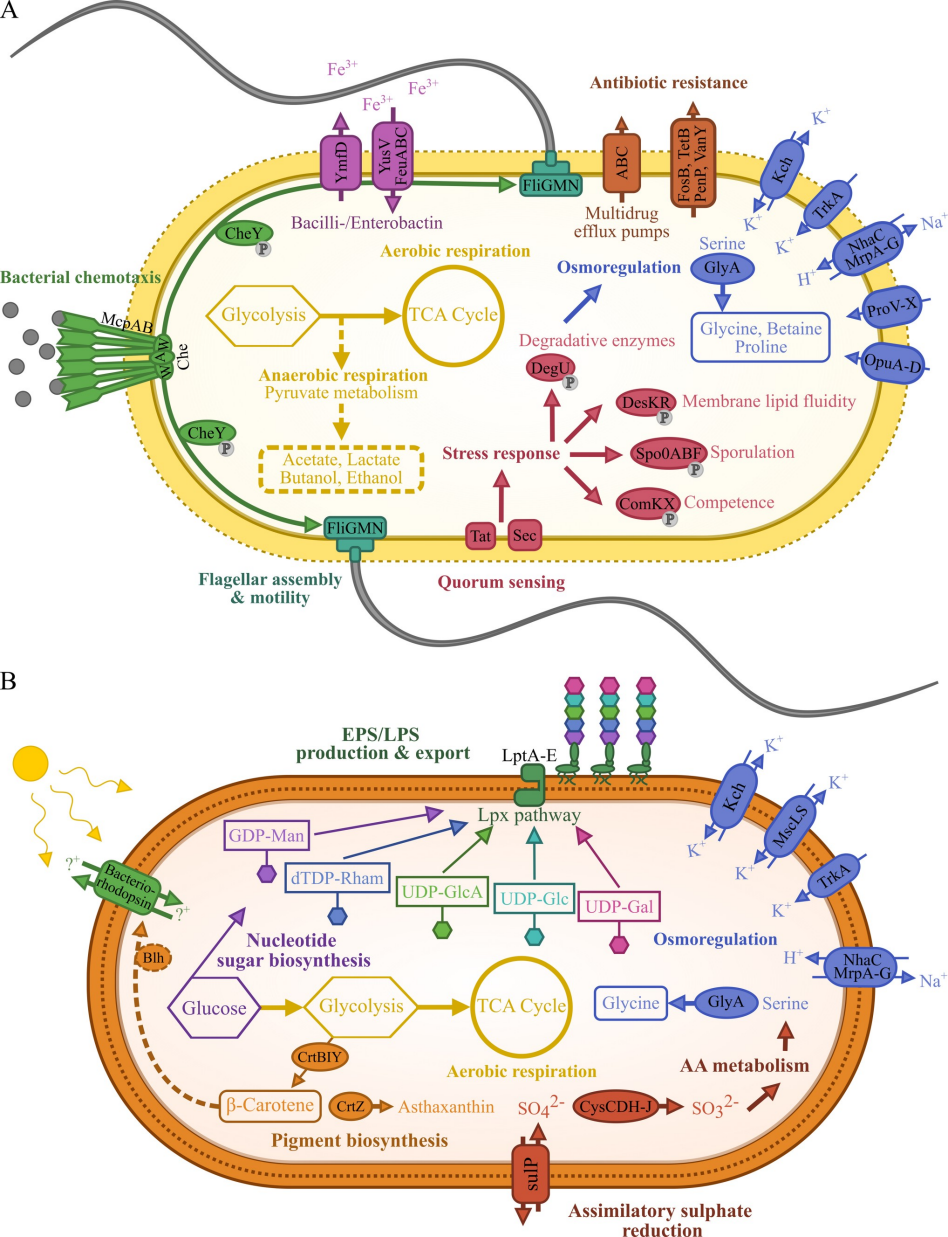
Key aspects of the *Pontibacillus* sp. ALD_SL1 (A) and *Psychroflexus* sp. ALD_RP9 (B) metabolism. Metabolic pathways and capabilities were reconstructed from the complete genomes against the KEGG database using BlastKoala [33] and the KEGG Mapper [34].

*Pontibacillus* sp. ALD_SL1 protein sequences were sorted into KEGG categories using BlastKoala [33] and KEGG Mapper [34], resulting in the assignment of 2,305 entries to 214 pathways (Table 3). Most genes of the *Pontibacillus* sp. ALD_SL1 genome were assigned to carbohydrate metabolism (266), followed by amino acid metabolism (228) and the metabolism of cofactors and vitamins (114). The most prevalent genes on the megaplasmid belong to cell motility (40) and replication and repair (32). *Pontibacillus* sp. ALD_SL1, like *P*. *litoralis* [3] and *P*. *salipaludis* [6], is putatively one of the facultatively anaerobic members of the genus. Under anaerobic conditions, it can generate energy potentially through oxidative phosphorylation (cox genes, F-type ATPase), and pyruvate metabolism, including fermentation to acetate (ala, pta, ackA), lactate (ldh), formate (pflD), butanol (crt, ptb, buk) and ethanol (ald, adh). Under nutrient-limiting conditions, ALD_SL1 can scavenge for iron using the siderophores bacilli- and enterobactin (entA-E). All 19 putative antibiotic resistance genes (ARGs) predicted for *Pontibacillus* sp. ALD_SL1 were located on the chromosome. The genes are mainly ATP-binding cassette (ABC) transporters, such as multidrug efflux pumps. In addition, putative resistance genes to fosfomycin (fosB), aminoglycosides (aacC, aadD, aadK), glycopeptides (vanY), tetracycline (tetB) and β-lactam antibiotics (penP) were detected (S2 Table). Resistance to aminoglycosides, tetracycline, phosphomycin and ampicillin has previously been shown for other isolates of the genus [3, 5, 6]. ABC transporters not only convey resistance to antibiotics, but also act as channels for osmotic regulation in the marine environment. Transporters include Na^+^/H^+^ antiporters (nhaC, mrpA-G) regulating cell homeostasis [44], K^+^ transporters (trkA, kch), as well as mechanosensitive channels (mscL) (Fig 5A). Genes for the transport of compatible solutes proline, glycine and betaine (proV-X, opuD/betL, opuA-C), which act as osmolytes [45], were detected as in other *Pontibacilli* [8]. Genes for ectoine synthesis, which are common in many halotolerant bacteria [45 and references therein] as well as *P*. *marinus* [4], were not detected in the ALD_SL1 genome. Further regulation of osmotic and other stressors could occur via the encoded putative quorum-sensing and subsequent phosphor-relay systems. These are involved in the regulation of membrane fluidity (desK/R), degradative enzymes (degS/U) [46], competence (comX/K) [47], and induce sporulation (spo genes) [48] (Fig 5A). While regulators of cell competence were present in the genome, a type IV secretion system for plasmid conjugation was not detected. ALD_SL1 can sense its environment and adjust its position using chemotaxis genes (mcp/che), which control flagella movement [49]. The chemotaxis receptors may be sensitive to oxygen, amongst other attractants, as the isolate showed aerotactic behaviour during motility testing. Most of its motility genes (i.e., fliG/M/N, motA/B) are situated both at the chromosome and the megaplasmid (Table 3). While encoding the same putative proteins, the genes share only low homology to each other. This indicates that they were acquired through horizontal gene transfer via the large mobile genetic elements (genomic islands/prophages) on the plasmid, rather than by interreplicon duplication [50]. As the plasmid does not harbour any essential genes, it is likely not critical for cell viability.

**Table 3:** Functional assignment of genes into KEGG categories.

KEGG category	*Pontibacillus* sp.		*Psychroflexus* sp.
	ALD_SL1		ALD_RP9
	Chromosome	Plasmid	Chromosome
Total Entries/Pathways	2,305/214	252/72	1,160/214
**Metabolism**			
Carbohydrate metabolism	266	8	157
Energy metabolism	101	1	90
Lipid metabolism	59	7	40
Nucleotide metabolism	73	9	65
Amino acid metabolism	228	10	179
Metabolism of other amino acids	38	4	26
Glycan biosynthesis and metabolism	34	9	49
Metabolism of cofactors and vitamins	114	7	97
Biosynthesis of other secondary metabolites	29	1	28
Xenobiotics biodegradation and metabolism	32	1	32
**Genetic Information Processing**			
Transcription	7	3	3
Translation	82	0	79
Folding, sorting and degradation	35	3	28
Replication and repair	72	32	64
**Environmental Information Processing**			
Membrane transport	97	4	28
Signal transduction	78	10	37
**Cellular Processes**			
Transport and Catabolism	9	0	7
Cell growth and death	12	7	11
Cellular community—prokaryotes	76	5	30
Cell motility	50	40	4
**Human Diseases**			
Drug resistance: antimicrobial	18	3	18

*Psychroflexus* sp. ALD_RP9 genes matched 1,160 KEGG entries which were assigned to 214 pathways. Most entries belonged to amino acid (179) and carbohydrate (157) metabolism, followed by the metabolism of cofactors and vitamins (97). ARG search indicated that *Psychroflexus* sp. ALD_RP9 harbours six potential antibiotic resistance genes. Four of these are multidrug ABC transporters, one a multidrug and toxic compound extrusion (MATE) family resistance protein and one a bicyclomycin multidrug efflux protein (S2 Table). It has previously been shown, that *Psychroflexi* are resistant to aminoglycosides, polyketide, and quinolone antibiotics [13, 20], but the identified ARGs cannot directly be linked to resistance against specific antibiotic compounds. *Psychroflexus* sp. ALD_RP9 employs a different strategy compared to *Pontibacillus* sp. ALD_SL1 to cope with the high salinity in its environment. To maintain turgor, it employs a series of Na^+^/H^+^ antiporters (NhaC/D, MrpA) [44, 51] and (mechanosensitive) ion transport channels (MscL/S, Kch) and proteins (TrkA) [52]. These may also support its ability to cope with the high pH tolerated. In addition, ALD_RP9 has an extensive exopolysaccharide layer surrounding the cells (Fig 1) and colonies have an almost jelly-like consistency. The EPS layer can protect from high salinity and pH values, as well as provide protection against desiccation [53]. ALD_RP9 forms its EPS layer by using a variety of nucleotide sugars and the Raetz pathway (lpx/waa genes). They are translocated and connected with the outer membrane via a lipopolysaccharide (LPS) transport system (lpt genes, Fig 5B) [54]. Acquisition of EPS/LPS genes has previously been observed in *Ps*. *torquis*, for which it was hypothesised that they support growth under psychrophilic conditions [42]. *Psychroflexus* sp. ALD_RP9 harbours some additional EPS genes within regions which are absent from all other strains (Fig 4). This suggests that ALD_RP9 has acquired additional EPS genes to support its survival. While EPS provides some desiccation protection, ALD_RP9 uses carotenoids (crtB/I/Y/Z genes) within its cell membrane to protect from irradiation and oxidative stress. The carotenoids can also be cleaved into retinal by Blh, which is required as co-factor for bacteriorhodopsin function [55]. In *Psychroflexus* members the bacteriorhodopsins most likely act as proton pumps that can drive ATP synthesis. This can be used to supplement their energy metabolism while under osmotic and other stress conditions [56, 57].

## Conclusions

We describe two novel bacterial species within the *Pontibacillus* and the *Psychroflexus* genus. *Pontibacillus* sp. ALD_SL1 is the third facultative anaerobe of the genus and first *Pontibacillus* to harbour a (mega-)plasmid. The plasmid contains genomic islands in large proportions and likely supports chemotaxis and motility but is not essential for cell viability. *Psychroflexus* sp. ALD_RP9 shows an enhanced capability to grow at high pH values in comparison to other *Psychroflexus* species. It clusters phylogenetically with members harbouring smallest genomes of the genus. Further, it contains genomic regions which are not present in other *Psychroflexi* and encode for additional genes involved in EPS synthesis. These may provide enhanced protection towards the moderately hypersaline conditions in its habitat. Taken together, both *Pontibacillus* sp. ALD_SL1 and *Psychroflexus* sp. ALD_RP9 are highly adapted to their environment but follow different strategies to support their survival.

## Supporting information

S1 Table Growth ranges of *Pontibacillus* sp. ALD_SL1 and *Psychroflexus* sp. ALD_RP9. Measurements are given in triplicate as OD_600_ for each isolate. The starting OD has been subtracted from the values to reflect active growth rates.
(XLSX)

S2 Table Genomic features of *Pontibacillus* sp. ALD_SL1 and *Psychroflexus* sp. ALD_RP9. The table includes selected KEGG hits, antibiotic resistance genes, genomic islands and phages, and their location on the genomes.
(XLSX)
